# Characterizing risky alcohol use, cigarette smoking, e-cigarette use, and physical inactivity among cancer survivors in the USA—a cross-sectional study

**DOI:** 10.1007/s11764-022-01245-5

**Published:** 2022-08-14

**Authors:** Jiyeong Kim, Theresa H. Keegan

**Affiliations:** 1 Department of Public Health Sciences, University of California, Davis, 1 Shields Avenue, Davis, CA 95616 USA; 2 Division of Hematology and Oncology, UC Davis Comprehensive Cancer Center, 4501 X Street, Suite 3016, Sacramento, CA 95817 USA

**Keywords:** Cancer survivors, Unhealthy behavior, Alcohol consumption, Cigarette smoking, E-cigarette use, Physical inactivity

## Abstract

**Purpose:**

Unhealthy lifestyle behaviors are associated with inferior health outcomes among cancer survivors, including increased mortality. It is crucial to identify vulnerable subgroups, yet investigations have been limited. Thus, this study aimed to examine sociodemographic and clinical characteristics associated with risky health behaviors among cancer survivors.

**Methods:**

We used national, cross-sectional survey data (Health Information National Trends Survey, HINTS 2017–2020) for 2579 cancer survivors. We calculated the prevalence of risky alcohol use, current cigarette smoking, e-cigarette use, and not meeting physical activity guidelines. We performed weighted logistic regression to obtain multivariable-adjusted odds ratios (OR) for the association between each unhealthy behavior with sociodemographic and clinical characteristics.

**Results:**

Overall, 25% showed risky alcohol use, 12% were current cigarette smokers, 3% were current e-cigarette users, and 68% did not meet physical activity guidelines. Cancer survivors who were males, non-Hispanic Whites or African Americans, without a college education, not married and with comorbidities or psychological distress were more likely to have unhealthy behaviors. Those with lung disease or depression were 2 times as likely to smoke cigarette or e-cigarettes and those with psychological distress were 1.6 times as likely to be physically inactive. Moreover, risky drinkers (OR = 1.75, 95% CI = 1.22–2.52) and e-cigarette smokers (OR = 16.40, 95% CI 3.29–81.89) were more likely to be current cigarette smokers.

**Conclusions:**

We identified vulnerable subpopulations of cancer survivors with multiple unhealthy lifestyle behaviors.

**Implications for Cancer Survivors:**

Our findings inform clinicians and program and policy makers of the subgroups of cancer survivors to target for multiple health behavior interventions.

**Supplementary Information:**

The online version contains supplementary material available at 10.1007/s11764-022-01245-5.

## Introduction

In 2020, an estimated 19.3 million cancer survivors were in the USA [1]. Along with the early detection, adjuvant therapy, advanced medical procedures, and development of new cancer therapeutics, the number of people living with cancer is expected to increase [2]. Health behaviors, including alcohol consumption, cigarette use, and physical inactivity, can play important roles in improving mental health and cognitive function, physical health status, and health-related quality of life for cancer survivors.

Alcohol use increases the risk of cancer recurrence [3] or developing secondary malignancies in cancer survivors [4–11]. Among almost 100,000 head and neck cancer survivors, 13% of secondary malignancies were considered alcohol-associated, although the relationship of post-diagnosis alcohol consumption with recurrence and survival has been found to be inconsistent in other cancer types [12–14]. In addition, cigarette use is often associated with inferior disease outcomes, including increased all-cause or cancer specific mortality, in cancer survivors [15–23]. Although e-cigarettes were introduced as an alternative to quit cigarette smoking, recent studies found that it increased cancer risk and facilitated cancer progression [24–27] and its use is increasing among cancer survivors [28]. Moreover, moderate physical activity is associated with improvements in cancer-specific as well as all-cause mortality across 11 different types of cancer [29], and decreases in cancer recurrence among breast, colon, and prostate cancer survivors [30–32]. Due to the importance of health promotion strategies in cancer survivors, guidelines have been established for this population [33–37]. However, multiple studies found that that many cancer survivors do not meet health behavior guidelines [4–6, 38, 39]. A recent large cohort study showed that 14% of breast cancer survivors consumed alcohol daily and 18% were current tobacco users [5]. Nearly 70% of cancer survivors did not achieve the physical activity guidelines in the USA, and up to 24% were current smokers [40].

Moreover, some studies identified subgroups of cancer survivors, defined by sociodemographic factors (e.g., age, sex), clinical status (e.g., psychological distress, cancer types, time since diagnosis), and treatment-related determinants [5, 41, 42], with unhealthy behaviors. For example, in a review, Demark-Wahnefried et al. reported male, older, less-educated, or urban residing cancer survivors were less likely to be successful at adopting and keeping healthy lifestyle behaviors [39]. Furthermore, previous studies reported the associations between multiple unhealthy behaviors in cancer survivors. For example, alcohol intake and physical inactivity were associated among breast cancer survivors [14].

However, limited studies have examined the health behaviors of cancer survivors by sociodemographic or clinical factors to specify subgroups with disparities [5, 14, 41, 42, 44, 46–50] and those that have considered these associations were often limited to certain cancer types (e.g., breast, thyroid, head and neck, prostate, colorectal, or gastric cancer) [5, 14, 38, 43, 45–48] or were conducted more than a decade ago when e-cigarette use was uncommon [14, 39, 44–47]. Identifying the subgroups of cancer survivors who are vulnerable to adopting and maintaining healthy lifestyles will be crucial to identify targeted populations for interventions to manage overall health and improve survival. Therefore, this study aims to examine unhealthy behaviors, including alcohol consumption, cigarette and e-cigarette use, and physical inactivity, among cancer survivors in the USA.

## Method

### Data source

The present study used publicly available, cross-sectional data from the Health Information National Trends Survey (HINTS) [49]. HINTS is a nationally representative survey collected by the National Cancer Institute. This study used HINTS 5 cycles 1, 2, 3, and 4 in 2017–2020. HINTS 5 is a single mode mailed survey using a two-stage sample design, except for cycle 3 that employed a push-to web pilot method in addition to the mailed survey. This study used remediated data for cycle 3 that was released in March 2021. The questionnaires were administered in non-institutionalized civilians aged 18 and older in the USA. Geographic addresses were stratified by two areas with high concentration of minority population or low concentration of minority population in HINTS 5 cycles 2, 3, and 4. HINTS 5 cycle 1 included one more stratification in geographic address, counties of Central Appalachia. Our study followed the Strengthening the Reporting of Observational Studies in Epidemiology (STROBE) guidelines [50]. The total number of survey respondents in this study was 16,092. 3285 from cycle 1, 3504 from cycle 2, 5438 from cycle 3, and 3865 from cycle 4. Each response rate was 32.4%, 32.4%, 30.3%, and 36.7%, respectively [49]. Among the total respondents, 2579 who reported a history of cancer diagnosis were included in this analysis. Data received full sample weights for the sample to be nationally representative. We assessed whether the variables were different across the cycles and the survey mode (mailed, web-based (paper return), web-based (web-returned)) prior merging the data. As no significant differences were identified in the variables of our interest by survey mode, we processed the data merging of all 4 cycles. Two hundred replicate weights were obtained by merging cycles 1 to 4 and applied to calculate standard errors as suggested by HINTS analysis recommendations. The full-sample weight accounted for household-level base weight, non-response, person-level initial weight, and other biases [51].

### Cancer survivor status

Consistent with the National Cancer Institute definition of cancer survivor [52], cancer survivor status in this study was identified by the question: “Have you ever been diagnosed as having cancer?” Those who affirmatively responded “yes” were defined as cancer survivor. Using the question, “At what age, were you first told you had a cancer?” HINTS calculated time since cancer diagnosis and provided it in 4 levels: less than 1 year, 2–5 years, 6–10 years, more than 11 years. Participants reported their cancer types and were classified as having breast, cervical, prostate, colon, lung, skin cancer, melanoma, multiple cancers, and other cancers. Other cancers included bladder, bone, endometrial, head and neck, leukemia/blood, liver, lymphoma (Hodgkin’s and non-Hodgkin’s), oral, ovarian, pancreatic, pharyngeal, rectal, renal, and stomach cancer.

### Outcome variables

#### Alcohol consumption

To investigate the number of average drinks per week, we used data derived from two survey questions about average number of drinks per day and the number of days of having at least one drink per week during the past 30 days. We categorized alcohol consumption per week into light (0–3), moderate (4–6), and heavy (≥ 7) drinks, as done previously [42, 53]. While there has been no consensus on the alcohol consumption guidelines for cancer survivors, we primarily referred to the National Health Interview Survey (NHIS) categories [53]. Then, we combined moderate and heavy drinking as risky alcohol use because American Cancer Society (ACS) and International Agency for Research on Cancer (IARC) both consider these high-risk groups [42, 54, 55].

#### Cigarette use

To investigate the current smoking status, the following questionnaire was used. “How often do you now smoke cigarettes?” Respondents who answered “Every day” and “Some days” were considered as current smokers. To investigate the current e-cigarette smoking status, the similar questionnaire was used. “Do you now use e-cigarette every day, some days, or not at all?” If respondents answered “Every day” or “Some days,” they were considered as current smokers or e-cigarette smokers, as done previously [42].

#### Physical activity

To investigate the weekly minutes of moderate exercise, data from two survey questionnaires about the number of days of moderate exercise (such as brisk walking, bicycling at a regular pace, and swimming at a regular pace) per week and minutes of moderate exercise per day were used. We categorized the level of physical activity into two groups: physically active (more than 150 min of weekly moderate exercise) and physically inactive (0–150 min of weekly moderate exercise) based on the US Physical Activity Guidelines [56, 57].

### Covariates

The conceptual framework of social determinants of health from the Healthy People 2030 [58] was applied for the choices of sociodemographic predictors in this study: age (18 to 34, 35 to 49, 50 to 64, 65 to 74, 75 or older), birth gender (male, female), race/ethnicity (non-Hispanic White, non-Hispanic Black/African American, Hispanic, non-Hispanic Asian, other), household income (< $20,000, $20,000 to < $35,000, $35,000 to < $50,000, $50,000 to < $75,000, $75,000 ≤), educational attainment (less than high school, high school graduate, some college, college graduate or more), marital status (married or living with a romantic partner as a married vs. not married including divorced, widowed, separated, single/never been married), employment status (employed vs. unemployed including homemaker, student, retired, disabled), health insurance type (insured by employment, private insurance, Medicaid, Medicare, Tricare, Veterans Affairs, Indian Health Services), rurality of residence (metropolitan, micropolitan, small town, rural). Rurality was determined by Urban Rural Commuting Area (RUCA) that categorizes census tracts based on population density, urbanization, and commuting patterns developed by US Department of Agriculture [59]. Clinical predictors included medical conditions (diabetes, high blood pressure, heart disease, lung disease, arthritis, depression) and psychological distress (little interest, hopelessness, nervousness, worrying).

### Statistical analysis

In descriptive analysis, we conducted a chi-square (categorical data) and a *t*-test (continuous data) to demonstrate demographic and clinical characteristics of cancer survivors and the prevalence of unhealthy behaviors (alcohol consumption, cigarette use, e-cigarette use, physical inactivity). Categorical data was presented with frequency (*n*) and weighted percentage (%) and continuous data was presented with mean and standard deviation (Tables 1 and 2).

**Table 1:** Sociodemographic and clinical characteristics of cancer population

	Frequency (n)		Weighted percentage (%)	Standard error (SE)
Sociodemographic characteristics				
Age (years)				
18–34	36		4.2	1.2
35–49	164		12.0	1.2
50–64	727		33.3	1.5
65–74	832		25.4	1.2
≥ 75	765		25.1	1.0
Missing (*n*)	55			
Gender				
Male	1065		43.1	1.5
Female	1487		57.0	1.5
Missing (*n*)	27			
Race/ethnicity				
White	1759		79.3	1.4
Black/African American	224		8.4	1.1
Hispanic	184		8.6	1.1
Asian	44		1.6	0.3
Others	70		2.1	0.4
Missing (*n*)	298			
Education				
Less than high school	152		6.8	1.0
High school graduate	535		27.2	1.4
Some college	780		37.8	1.5
College graduate or more	1058		28.1	1.2
Missing (*n*)	54			
Household income				
< $20,000	414		16.9	1.4
$20,000 to < $35,000	362		14.5	1.1
$35,000 to < $50,000	324		15.7	1.6
$50,000 to < $75,000	415		18.8	1.4
≥ $75,000	727		34.1	1.6
Missing (*n*)	337			
Marital status				
Married	1310		61.7	1.5
Not married	1215		38.3	1.5
Missing (*n*)	54			
Employment^**#**^				
Employed	507		35.9	1.8
Not employed	1163		64.1	1.8
Missing (*n*)	909			
Area: rurality				
Metropolitan	2186		81.9	1.1
Micropolitan	212		10.3	0.9
Small town	98		3.9	0.6
Rural	83		3.9	0.5
Missing (*n*)	0			
Health insurance				
Employment and private	654		35.1	1.6
Medicare	1165		38.9	1.4
Medicaid	300		15.6	1.4
Tricare, VA, IHS	316		10.4	0.8
Missing (*n*)	144			
Clinical characteristics				
Medical condition	Frequency, *N*	Missing (*n*)		
Diabetes	693	57	24.3	1.4
High blood pressure	1492	49	54.4	1.5
Heart disease	409	33	15.1	1.1
Lung disease	452	38	16.6	1.0
Arthritis^#^	487	1493	38.9	2.1
Depression	579	55	22.7	1.2
Psychological distress	Frequency, *N*	Missing (*n*)		
Little interest	850	55	35.8	1.5
Hopelessness	712	62	30.0	1.5
Nervousness	868	55	37.8	1.6
Worrying	753	55	32.8	1.6
Time since diagnosis				
< 1 year	304		13.9	1.2
2–5 years	513		20.6	1.2
6–10 years	460		18.5	1.2
≥ 11 years	1148		47.0	1.6
Missing (*n*)	154			
Cancer type				
Breast cancer	370		13.2	1.0
Cervical cancer	132		6.8	0.9
Prostate cancer	234		6.5	0.6
Colon cancer	106		3.9	0.5
Skin cancer	635		24.7	1.3
Melanoma	118		5.0	0.7
Lung cancer	49		1.8	0.4
More than one cancer	438		16.6	1.0
Other cancer	453		21.4	1.6
Missing (*n*)	44			

*VA*, Veterans Affairs; *IHS*, Indian Health Services

^#^Data is missing in some cycles: arthritis is not reported in cycles 3 and 4, employment is not reported in cycle 3. Missing frequency: arthritis *n* = 1473, employment *n* = 909

**Table 2:** Prevalence of unhealthy behaviors by sociodemographic and clinical factors among cancer survivors^+^

	Risky alcohol use weighted % (SE) *N* = 1827^#^		Cigarette use weighted % (SE) *N* = 2556	E-cig use weighted % (SE) *N* = 2547	Physical inactivity weighted % (SE) *N* = 2508
	4–6 drinks	7 ≤ drinks	Current	Current	0–150 min/wk
Total	8.7 (1.0)	16.2 (1.4)	11.8 (1.1)	2.9 (0.6)	68.2 (1.6)
Sociodemographic characteristics					
Age (years)					
18–34	4.1 (2.7)	2.7 (2.2)	9.2 (6.6)	0 (0)	82.7 (8.5)*
35–49	13.4 (4.3)*	15.5 (5.1)*	18.7 (5.0)*	6.7 (2.7)*	66.6 (5.8)
50–64	10.8 (2.0)*	17.2 (2.5)*	15.1 (1.9)*	2.1 (0.8)	65.2 (2.7)
65–74	8.5 (1.5)	16.1 (2.1)	11.3 (1.7)	2.8 (1.1)	64.4 (2.4)
≥ 75	4.3 (1.0)	15.8 (2.2)	4.3 (1.0)	1.8 (0.8)	75.2 (2.1)*
Gender					
Male	8.6 (1.5)*	22.0 (2.1)*	10.8 (1.7)	1.9 (0.8)	65.9 (2.5)
Female	8.7 (1.3)	11.8 (1.7)	12.5 (1.3)*	3.7 (0.9)*	69.7 (2.1)*
Race/ethnicity					
White	9.9 (1.3)*	18.4 (1.8)*	12.1 (1.3)*	3.5 (0.8)*	65.5 (1.9)
Black/African American	2.3 (1.3)	7.4 (2.8)	10.1 (4.2)	0.2 ( 0.2)	81.8 (4.9)*
Hispanic	2.8 (2.1)	7.7 (3.1)	6.8 (2.0)	4.0 (1.9)*	75.0 (6.6)*
Asian	N/A	14.3 (12.6)	3.6 (2.5)	0 (0)	75.3 (9.5)*
Others	6.7 (5.7)	9.4 (5.2)	19.5 (8.1)*	0.6 (0.5)	81.9 (6.8)*
Education					
Less than high school	7.0 (3.8)	6.8 (3.3)	9.0 (3.1)	6.2 ( 3.3)*	86.3 (4.8)*
High school graduate	5.3 (1.7)	13.1 (2.8)	19.7 (2.7)*	2.7 (1.1)	73.8 (2.9)*
Some college	7.8 (1.7)	14.4 (2.0)	11.1 (1.7)	4.0 (1.2)*	68.2 (2.5)
College graduate or more	13.2 (1.6)*	23.3 (2.4)*	5.7 (0.9)	1.1 (0.4)	58.2 (2.1)
Household income					
< $20,000	5.5 (1.9)	7.5 (2.3)	21.1 (3.3)*	5.9 (2.0)*	77.0 (4.1)*
$20,000 to < $35,000	7.2 (2.9)	11.4 (2.6)	11.8 (2.4)	3.2 (1.4)*	75.4 (3.2)*
$35,000 to < $50,000	5.1 (2.5)	15.4 (2.9)	15.2 (2.8)*	3.4 (1.9)*	73.1 (4.4)*
$50,000 to < $75,000	9.3 (2.1)*	22.0 (4.2)*	16.8 (3.8)*	4.8 (2.1)*	63.6 (3.8)
≥ $75,000	12.4 (2.2)*	22.6 (2.9)*	3.4 (0.8)	0.7 (0.3)	59.4 (2.7)
Marital status					
Married	8.7 (1.3)*	17.4 (2.0)*	8.8 (1.3)	1.6 (0.5)	66.4 (2.1)
Not married	8.6 (1.6)	14.3 (1.7)	15.3 (1.9)*	4.5 (1.3)*	71.4 (2.4)*
Employment					
Employed	11.8 (2.8)*	17.3 (3.0)*	12.9 (2.5)*	3.7 (1.4)*	64.8 (3.1)
Not employed	5.7 (1.1)	13.4 (2.0)	10.9 (1.5)	2.6 (0.8)	69.6 (2.2)*
Area: rurality					
Metropolitan	9.0 (1.1)*	17.7 (1.6)*	12.4 (1.2)*	3.1 (0.7)*	68.1 (1.7)
Micropolitan	4.4 (1.9)	7.2 (3.2)	9.9 (2.6)	0.3 (0.2)	69.2 (5.5)*
Small town	6.6 (4.6)	12.9 (7.2)	7.4 (2.9)	2.6 (2.8)	60.9 (8.0)
Rural	15.0 (6.2)	9.2 (4.3)	7.9 (3.5)	6.7 (4.5)*	73.9 (5.8)*
Health insurance					
Employment and private	14.0 (2.2)*	19.1 (2.8)*	13.2 (2.3)*	2.6 (1.1)	58.6 (3.0)
Medicare	7.7 (1.2)	15.3 (1.8)	7.1 (1.0)	1.7 (0.6)	69.8 (1.9)*
Medicaid	4.6 (2.0)	6.0 (2.3)	17.0 (3.0)*	6.9 (2.4)*	81.2 (3.5)*
Tricare, VA, IHS	3.0 (1.3)	21.1 (4.2)	9.3 (3.4)	1.2 (0.6)	73.9 (3.3)*
Clinical characteristics					
Medical condition					
Diabetes	5.1 (2.1)	9.4 (1.8)	10.6 (1.6)	3.1 (0.9)*	73.2 (2.9)*
High blood pressure	6.5 (1.1)	16.9 (1.8)	11.1 (1.3)	2.7 (0.7)	71.6 (2.0)*
Heart disease	5.9 (1.7)	15.5 (3.5)	11.3 (2.5)	1.9 (0.9)	78.1 (3.3)*
Lung disease	7.0 (1.9)	10.0 (2.7)	18.1 (2.7)*	6.6 (2.4)*	77.4 (3.0)*
Arthritis	2.7 (1.1)	13.7 (3.4)	9.5 (1.7)	3.0 (1.3)*	73.6 (2.9)*
Depression	8.7 (2.1)	13.0 (2.4)	23.2 (2.9)*	7.0 (1.7)*	76.7 (2.5)*
Psychological distress					
Little interest	8.0 (1.9)	15.2 (2.8)	16.7 (2.1)*	5.5 (1.4)*	78.5 (2.6)*
Hopelessness	9.8 (2.1)*	16.7 (3.2)*	17.4 (2.3)*	5.8 (1.5)*	76.7 (2.8)*
Nervousness	9.4 (2.0)*	16.3 (2.7)*	16.4 (2.0)*	6.0 (1.4)*	74.2 (2.6)*
Worrying	9.4 (2.1)	15.0 (2.8)	16.2 (2.3)*	6.5 (1.7)*	73.4 (2.9)*
Time since diagnosis					
< 1 year	5.6 (2.0)	9.7 (2.8)	6.2 (1.4)	2.3 (1.1)	71.8 (4.2)*
2–5 years	7.8 (2.2)*	21.9 (3.5)*	13.9 (2.9)*	3.0 (1.4)*	67.1 (3.0)
6–10 years	13.3 (2.6)*	16.0 (3.6)*	11.9 (2.7)*	3.1 (1.5)*	64.7 (3.5)
≥ 11 years	8.5 (1.5)	15.2 (1.8)	11.8 (1.5)	2.8 (0.9)	69.4 (2.3)*
Cancer type					
Breast cancer	8.6 (2.3)*	17.1 (4.8)*	13.9 (3.2)*	3.9 (1.6)*	69.7 (4.0)*
Cervical cancer	16.3 (6.7)	5.0 (2.4)	20.3 (5.3)*	4.0 (2.4)*	71.4 (7.0)*
Prostate cancer	5.4 (2.2)*	24.5 (5.1)*	9.1 (3.1)	2.7 (2.7)	64.7 (4.9)
Colon cancer	3.8 (2.2)	11.9 (4.6)	14.5 (8.0)*	7.3 (7.5)	75.5 (8.1)*
Skin cancer	9.7 (1.9)*	21.8 (2.6)*	7.8 (1.7)	1.1 (0.4)	62.3 (2.8)
Melanoma	5.7 (2.5)*	26.6 (8.8)*	13.5 (6.7)*	1.2 (0.9)	78.6 (5.4)*
Lung cancer	4.4 (4.0)	16.0 (9.6)	22.2 (12.0)*	14.6 (11.9)*	67.9 (9.6)
More than one cancer	8.4 (2.4)	16.2 (3.0)	11.9 (2.6)*	3.8 (1.6)*	70.4 (3.1)*
Other cancer	8.7 (2.2)	8.8 (2.0)	11.8 (2.2)	2.2 (0.8)	69.1 (4.0)*

^*^Higher than the average prevalence

^+^Full information of prevalence of health behaviors can be found in supplementary file 1

Abbreviations. *VA*, Veterans Affairs; *IHS*, Indian Health Services

^#^Alcohol consumption is not collected in cycle 1

Some variables were not available from all 4 cycles; alcohol consumption was not reported in cycle 1, arthritis was not reported in cycles 3 and 4, and employment was not reported in cycle 3. Survey weighted multivariate logistic regression was performed to estimate the odds of alcohol consumption (light, moderate/heavy drinking), cigarette use (current, former, never), e-cigarette use (current, former, never), and physical activity (0–150, 150 < weekly minutes) for selected sociodemographic factors (e.g., age, birth gender, race/ethnicity, educational attainment, household income, marital status, health insurance, rurality of residence) and clinical covariates (e.g., medical condition and psychological distress) (Table 3). Due to small number of cancer survivors aged 18–34 years, they were combined with the 35–49 years age group. Employment and arthritis were not included in the weighted logistic regression analysis due to the large proportion missing (35% and 52%, respectively). Unadjusted and fully adjusted analyses for independent variables across all our unhealthy behavior outcomes were conducted. When compared, variables that changed the effect estimate by more than 10% were kept in the final multivariable model (Table 3). We also performed weighted survey logistic regression for each health behavior to observe relationships between multiple unhealthy behaviors after adjusting associated factors found in Table 3 (Table 4). For the weighted survey logistic regression analyses, we used a complete case analysis. The statistical significance was determined at *p* value < 0.05. SAS 9.4 (SAS studio 3.8, Cary, NC, USA) was used for the analysis.

**Table 3:** Associations of sociodemographic and clinical factors with unhealthy behaviors among cancer survivors

	Risky alcohol use *N* = 1355^#^	Current cigarette use *N* = 1836	Current e-cig use *N* = 1834	Physical inactivity *N* = 1813
	OR^+^ (95% CI)	OR^+^ (95% CI)	OR^+^ (95% CI)	OR^+^ (95% CI)
Age (years)				
18–49	1.09 (0.45, 2.67)	0.66 (0.29, 1.49)	6.84 (1.35, 34.70)*	1.07 (0.49, 2.35)
50–64	0.87 (0.38, 2.00)	0.81 (0.47, 1.40)	2.97 (0.78, 11.29)	0.96 (0.50, 1.84)
65–74	1.38 (0.83, 2.31)	1.26 (0.90, 1.75)	2.65 (1.04, 6.72)*	0.61 (0.42, 0.89)*
≥ 75	Reference	Reference	Reference	Reference
Gender				
Male	1.87 (1.10, 3.16)*	1.54 (1.07, 2.11)*	1.63 (0.85, 3.16)	0.94 (0.66, 1.34)
Female	Reference	Reference	Reference	Reference
Race/ethnicity				
White	Reference	Reference	Reference	Reference
Black/African American	0.28 (0.11, 0.74)*	0.44 (0.21, 0.93)*	0.13 (0.02, 0.73)*	2.17 (1.05, 4.48)*
Hispanic	0.23 (0.09, 0.65)*	0.64 (0.32, 1.27)	0.83 (0.33, 2.12)	0.97 (0.38, 2.44)
Asian	0.29 (0.01, 10.54)	0.35 (0.08, 1.58)	0.31 (0.12, 0.79)*	2.45 (0.61, 9.88)
Others	0.49 (0.10, 2.50)	1.20 (0.42, 3.44)	0.37 (0.07, 2.13)	2.09 (0.72, 6.07)
Education				
Less than high school	1.06 (0.33, 3.37)	1.63 (0.69, 3.85)	4.48 (1.01, 19.82)*	2.09 (0.49, 9.00)
High school graduate	0.57 (0.27, 1.19)	1.96 (1.18, 3.26)*	3.08 (1.31, 7.25)*	1.49 (0.89, 2.52)
Some college	0.75 (0.46, 1.23)	1.88 (1.36, 2.61)*	2.15 (1.09, 4.23)*	1.49 (1.01, 2.20)*
College graduate or more	Reference	Reference	Reference	Reference
Household income				
< $20,000	Reference	Reference	Reference	Reference
$20,000 to < $35,000	1.15 (0.45, 2.92)	0.52 (0.27, 1.03)	0.82 (0.30, 2.25)	0.96 (0.49, 1.86)
$35,000 to < $50,000	1.43 (0.58, 3.54)	0.71 (0.32, 1.57)	0.87 (0.29, 2.58)	1.00 (0.49, 1.86)
$50,000 to < $75,000	2.00 (0.79, 5.04)	0.98 (0.47, 2.03)	1.39 (0.46, 4.21)	0.92 (0.45, 1.86)
≥ $75,000	2.11 (0.89, 5.02)	0.57 (0.27, 1.23)	0.71 (0.22, 2.29)	1.04 (0.50, 2.13)
Marital status				
Married	Reference	Reference	Reference	Reference
Not married	1.53 (0.91, 2.58)	1.24 (0.86, 1.79)	2.00 (1.00, 4.00)*	1.01 (0.70, 1.45)
Area: rurality				
Metropolitan	Reference	Reference	Reference	Reference
Micropolitan	0.31 (0.12, 0.82)	0.60 (0.38, 0.97)*	0.31 (0.14, 0.69)*	1.34 (0.68, 2.65)
Small town	0.71 (0.19, 2.66)	0.53 (0.18, 1.54)	0.37 (0.04, 3.85)	0.53 (0.21, 1.37)
Rural	0.71 (0.22, 2.29)	1.06 (0.59, 1.90)	1.11 (0.24, 5.15)	2.13 (1.01, 4.46)*
Health insurance				
Employment and private	Reference	Reference	Reference	Reference
Medicare	0.41 (0.21, 0.82)*	0.78 (0.43, 1.41)	0.70 (0.22, 2.25)	1.88 (1.08, 3.28)*
Medicaid	0.28 (0.10, 0.79)*	0.58 (0.27, 1.21)	0.66 (0.23, 1.89)	2.16 (1.03, 4.55)*
Tricare, VA, IHS	0.42 (0.17, 1.02)	0.94 (0.49, 1.82)	0.83 (0.29, 2.39)	1.99 (1.09, 3.62)*
Medical condition				
Heart disease (vs. none)	1.00 (0.49, 2.04)	1.35 (0.94, 1.95)	0.41 (0.18, 0.96)*	1.27 (0.74, 2.21)
Lung disease (vs. none)	0.97 (0.54, 1.74)	1.65 (1.12, 2.41)*	2.25 (1.11, 4.57)*	1.06 (0.69, 1.62)
Depression (vs. none)	0.77 (0.46, 1.30)	1.95 (1.25, 3.03)*	2.26 (1.12, 4.56)*	1.33 (0.89, 2.01)
Psychological distress				
Little interest (vs. none)	1.19 (0.71, 2.01)	1.33 (0.92, 1.92)	1.80 (0.93, 3.51)	1.59 (1.01, 2.49)*

^**+**^Adjusted for all the variables in the table (age, gender, race/ethnicity, education, income, marital status, rurality, health insurance, medical condition, psychological distress)

^*^p value < 0.05

^#^Alcohol consumption was not collected in cycle 1

*VA*, Veterans Affairs; *IHS*, Indian Health Services

**Table 4:** Associations between unhealthy behaviors among cancer survivors

Health behaviors	Risky alcohol use^**+**^ *N* = 1458	Current cigarette use^**++**^ *N* = 1554	Current e-cig use^**#**^ *N* = 1545	Physical inactivity^**##**^ *N* = 1507
	OR (95% CI)	OR (95% CI)	OR (95% CI)	OR (95% CI)
Alcohol use				
Light drinkers	N/A	Reference	Reference	Reference
Moderate/heavy drinkers	N/A	1.75 (1.22, 2.52)^*^	1.00 (0.49, 2.03)	0.56 (0.38, 0.84)^*^
Cigarette use				
Current	2.12 (0.95, 4.74)	N/A	58.39 (18.46, 184.66)^*^	1.41 (0.63, 3.19)
Former	1.58 (1.04, 2.41)^*^	N/A	14.82 (4.76, 46.20)^*^	1.17 (0.73, 1.86)
Never	Reference	N/A	Reference	Reference
E-cigarette use				
Current	1.58 (0.39, 6.35)	16.40 (3.29, 81.89)^*^	N/A	0.43 (0.06, 3.08)
Former	1.01 (0.40, 2.55)	10.19 (4.70, 22.13)^*^	N/A	2.00 (0.65, 6.12)
Never	Reference	Reference	N/A	Reference
Physical activity				
0–150 min	Reference	Reference	Reference	N/A
> 150 min	1.74 (1.15, 2.63)^*^	0.90 (0.59, 1.37)	0.57 (0.23, 1.40)	N/A

^**+**^Adjusted for other health behaviors in the table (cigarette use, e-cigarette use, physical activity) and gender, race/ethnicity, and health insurance

^**++**^Adjusted for other health behaviors in the table (alcohol use, e-cigarette use, physical activity) and gender, education, race/ethnicity, rurality, lung disease, and depression

^**#**^Adjusted for other health behaviors in the table (alcohol use, cigarette use, physical activity) and age, race/ethnicity, education, marital status, rurality, heart disease, lung disease, and depression

^**##**^Adjusted for other health behaviors in the table (alcohol use, cigarette use, e-cigarette use) and age, race/ethnicity, education, rurality, health insurance, and psychological distress

^*^p value < 0.05

## Results

### Cancer survivor characteristics

Among the 2579 cancer survivors in our study, 84% were 50 years or older (Table 1). Cancer survivors were more likely to be females (57%) than males, non-Hispanic Whites (79%) than Black/African Americans (8.4%), Asians (1.6%), and others (2.1%) and married (62%) than unmarried. More than half of cancer survivors had some college education or more (65%). Nearly half of cancer survivors reported their household income was less than $50,000 (47%), and they had psychological distress (48%), including little interest, hopelessness, nervousness, or worrying. Among those, the prevalence of each psychological distress was approximately 30–38%. High blood pressure and arthritis were 2 most common comorbidities among cancer survivors, 54.4% and 38.9%, respectively. Half of cancer survivors have been diagnosed with cancer for 11 years or longer (47%) while 14% were for less than 1 year. Skin (24.7%), breast (13.2%), and more than one cancer (16.6%) were the most prevalent diagnoses in this study.

### Prevalence of unhealthy behaviors

Most (75.1%, SE 1.7) cancer survivors were light drinkers, yet 16.2% were heavy drinkers. Median (interquartile range, IQR) of average drinks per week was 0 (4) and mean (standard deviation, SD) was 3.41 (7.76). The prevalence of risky alcohol use (moderate/heavy drinking) was greater than the average in some sociodemographic groups, including cancer survivors who were males (30.6%), were of non-Hispanic White race/ethnicity (28.3%), had college graduate or more education (36.5%), had household income $50,000 or more (31.3–35%), or in some clinical subgroups, including cancer survivors who were diagnosed with breast, prostate, skin cancer, or melanoma (25.7–32.3%) (Table 2). The full prevalence information of cancer survivors’ health behaviors can be found in supplementary file 1.

The majority (68.2%, SE 1.6) of cancer survivors reported that they exercised for 150 min or less than weekly in this study. Median (IQR) of weekly minutes of moderate exercise was 90 (180) and mean (SD) was 159.42 (295.71). The prevalence of physical inactivity was greater than the average in cancer survivors who were of Black/African American race/ethnicity (81.8%) or had high school or less education (73.8–86.3%), had Tricare/VA/IHS (73.9%). Cancer survivors with any comorbidities examined in this study (71.6–78.1%), had any type of psychological distress (73.4–78.5%), or were diagnosed with breast, cervical, colon, melanoma, more than one, or other cancer (69.1–78.6%) were more likely to be current e-cigarette smokers.

Fewer (11.8%, SE 1.1) cancer survivors were current cigarette smokers than former (34.0%, SE 1.3) or never smokers (54.3%, SE 1.3). The prevalence of current cigarette smoking was greater than the average in cancer survivors who were 35–64 years (15.1–18.7%), were females (12.5%), were of White (12.1%) race/ethnicity, had high school graduate education (19.7%), or were not married (15.3%). Current cigarette smoking was higher among those who had lung disease (18.1%), had depression (23.2%), had any type of psychological distress (16.2–17.4%), or were diagnosed with breast, cervical, colon, lung, more than one cancer, or melanoma (11.9–22.2%).

Only 2.9% (SE 0.6) were current e-cigarette users, while 89.4% (SE 1.0) were never e-cigarette smokers. The prevalence of current e-cigarette smoking was greater than the average in cancer survivors who were 35–49 years (6.7%), females (3.7%), of non-Hispanic White (3.5%) or Hispanic (4.0%) race/ethnicity, less educated, not married (4.5%), or metropolitan residents (3.1%). Current e-cigarette smoking was higher in those who had lung disease (6.6%), had depression (7.0%), had any type of psychological distress (5.5–6.5%), or were diagnosed with breast, cervical, lung, or more than one cancer (3.8–14.6%).

### Sociodemographic and clinical characteristics associated with unhealthy behaviors

Males had nearly 2 times the odds of risky drinking compared to females (OR = 1.87, 95% CI = 1.10–3.16). Black/African Americans had a lower odds of being risky drinkers than non-Hispanic White cancer survivors (OR = 0.28, 95% CI = 0.11–0.74). Cancer survivors with Medicaid or Medicare insurance had a lower odds of being risky drinkers as those with private insurance (OR = 0.28, 95% CI = 0.10–0.79, OR = 0.41, 95% CI = 0.21–0.82) (Table 3). Physically active cancer survivors were nearly 2 times as likely to have risky drinking behaviors as their non-active counterparts (OR = 1.74, 95% CI = 1.15–2.63 in 150 < vs. 0–150 min). Former cigarette smokers had 1.5 times the odds of risky drinking compared to never smokers (OR = 1.58, 95% CI = 1.04–2.41) (Table 4).

Males had 1.5 times the odds of smoking cigarette compared to females (OR = 1.54, 95% CI = 1.07–2.11). Non-Hispanic Whites were 2 times as likely to be current smokers as Black/African Americans (OR = 0.44, 95% CI = 0.21–0.93). Cancer survivors with less education (high school graduate/some college) had 2 times the odds of using cigarette currently compared to those with college graduate or more education (OR = 1.96, 95% CI = 1.18–3.26, OR = 1.88, 95% CI = 1.36–2.61). Metropolitan residents had a higher odds of smoking cigarettes as micropolitan residents (OR = 0.60, 95% CI = 0.38–0.97 in micropolitan vs. metropolitan). Cancer survivors with lung disease or depression were 1.5–2 times as likely to be current cigarette smokers as those without these diseases (OR = 1.65, 95% CI = 1.12–2.41, OR = 1.95, 95% CI = 1.25–3.03) (Table 3). Current/former e-cigarette smokers had 10–15 times the odds of using cigarette currently compared to never e-cigarette users (OR = 16.40, 95% CI = 3.29–81.89 in current e-cigarette users, OR = 10.19, 95% CI = 4.70–22.13 in former vs. never e-cigarette users). Moderate/heavy drinkers were nearly 2 times as likely to smoke cigarette as light drinkers (OR = 1.75, 95% CI = 1.22–2.52) (Table 4).

Younger cancer survivors (18–49 years) had 7 times the odds of using e-cigarette currently compared to the oldest survivors (≥ 75 years) (OR = 6.84, 95% CI = 1.35–34.70). Non-Hispanic Whites were 3–7 times as likely to be current e-cigarette users as Asians and Black/African Americans (OR = 0.31, 95% CI = 0.12–0.79 in Asians, OR = 0.13, 95% CI = 0.02–0.73 in Black/African Americans). Similar to the cigarette smoking, cancer survivors with less than college graduate had 2–4.5 times the odds of smoking e-cigarette currently compared to those with college graduate or more education (OR = 2.15, 95% CI = 1.09–4.23, OR = 3.08, 95% CI = 1.31–7.25, OR = 4.48, 95% CI = 1.01–19.82). Unmarried cancer survivors and metropolitan residents had 2–3 times the odds of smoking e-cigarette currently compared to their counterparts, married or micropolitan residents (OR = 2.00, 95% CI = 1.00–4.00 in unmarried, OR = 0.31, 95% CI = 0.14–0.69 in micropolitan, respectively). Cancer survivors with lung disease or depression were 2 times as likely to use e-cigarette as those without these health conditions (OR = 2.25, 95% CI = 1.11–4.57) (Table 3). Current/former cigarette users were significantly more likely to use e-cigarette than never cigarette smokers (OR = 58.39, 95% CI = 18.46–184.66 in current, OR = 14.82, 95% CI = 4.76–46.20 in former vs. never cigarette smokers) (Table 4).

Black/African Americans had 2 times the odds of being physically inactive compared to non-Hispanic White cancer survivors (OR = 2.17, 95% CI = 1.05–4.48). Cancer survivors with less education (some college) were 1.5 times as likely to be physically inactive as those with at least a college degree (OR = 1.49, 95% CI = 1.01–2.20). Rural residents had 2 times the odds of being physically inactive compared to metropolitan residents (OR = 2.13, 95% CI = 1.01–4.46). Publicly insured cancer survivors (Medicare, Medicaid, Tricare/VA/IHS) were 2 times as likely to be physically inactive as those with private insurance (OR = 1.88, 95% CI = 1.08–3.28, OR = 2.16, 95% CI = 1.03–4.55, OR = 1.99, 95% CI = 1.09–3.62). Cancer survivors with psychological distress (little interest) were 1.5 times as likely to be physically inactive as their counterparts (OR = 1.59, 95% CI = 1.01–2.49) (Table 3). Moderate/heavy drinkers had a lower odds of being physically inactive than light drinkers (OR = 0.56, 95% CI = 0.38–0.84) (Table 4).

## Discussion

Using nationally representative data, we identified sociodemographic and clinical subgroups of cancer survivors who have risky health behaviors, including males and those of non-Hispanic White and Black/African American race/ethnicity, without a college degree, not married, and with comorbidities or psychological distress. Overall, 24.9% of cancer survivors had risky alcohol use behaviors, 11.8% were current cigarette smokers, 2.9% currently used e-cigarettes, and 68.2% did not meet recommended physical activity guidelines. Compared to a recent evaluation of health behaviors in 12,648 cancer survivors (2013–2017) [42], our estimates are similar for current cigarette/e-cigarette smoking, but higher for moderate/heavy drinking and lower for not meeting physical activity guidelines. Lastly, we found associations with risky health behaviors, including unhealthy alcohol use, cigarette and e-cigarette smoking, unhealthy behaviors that have been found to cluster together in the general US population [60]. The findings of this study improve our understanding in cancer survivors’ health behaviors and contribute to designing effective and efficient behavior modification interventions.

We observed that current cigarette smokers and e-cigarette users shared common sociodemographic and clinical characteristics. Cancer survivors who were non-Hispanic White, less educated, not married, metropolitan residents, and with lung disease or depression were more likely to use both cigarette and e-cigarette. Our findings of an association between current cigarette use and e-cigarette use among cancer survivors are well aligned with the findings of multiple studies where e-cigarette use was associated with current cigarette smoking among the general population [61–64]. Thus, strategic cessation interventions to incorporate cigarette smokers, e-cigarette smokers, and dual smokers among cancer survivors will need to be considered. Additionally, the association of current drinking and current cigarette smoking that we observed in our study was found in multiple previous studies [41, 43, 65]. Moreover, in a prior study, current cigarette smoking status was related to physical inactivity among breast cancer patients, although the association was not significant in this study [5]. Overall, our findings suggest that interventions should consider addressing multiple risky health behaviors in cancer survivors.

We observed that cancer survivors with comorbidities and mental health conditions were more likely to smoke. Those with lung disease or depression were more likely to either cigarette or e-cigarette smoke. Moreover, cancer survivors with poor mental health (depression and little interest) were more likely to use e-cigarette and exercise less. The associations of poor mental health status with unhealthy behaviors were reported in prior studies, with the odds of risky alcohol use higher in breast cancer patients who were at-risk of depression [14] and the odds of e-cigarette smoking higher in cancer survivors experiencing depression [66]. Comorbidities and mental distress have been found to be more common among cancer survivors than adults without an history of cancer [67, 68], highlighting the need to target these populations for smoking cessation and physical activity interventions to improve outcomes. In particular, moderate aerobic fitness and strength training have been found to be associated with improvements in breast cancer survivors’ psychological state, including health-related quality of life, depression, anxiety, and fatigue [4, 69–71].

In our study, cancer survivors who were males or not married consistently presented with unhealthy drinking and smoking behaviors. Our results are well aligned with previous reports that found male cancer survivors to be less likely successfully adopt or maintain healthy lifestyles [39]. Moreover, individuals who had a non-smoking spouse were more likely to attempt to quit or be successful in tobacco product smoking [72, 73]. Previous studies also report that marriage was a significant transitional moment in reduction of risky alcohol use and drinking problems became moderate after marriage [74]. Given these findings, incorporating spouses or partners in behavior change programs should be considered for cancer survivors [72].

In our study, cancer survivors with less than a college degree were more likely to smoke cigarette or e-cigarettes and be physically inactive. The association of education with current cigarette smoking is consistent with prior studies of smoking cessation attempts and sustained cessation in the general population [62, 75]. These studies found that those with more education were more likely to attempt to quit smoking as well as to maintain cessation status than their less educated counterparts [62, 75]. Similarly, our findings of cancer survivors with lower education being more likely to be physically inactive are aligned with the previous studies among breast cancer survivors [5]. The association of lower education with a higher odds of multiple unhealthy behaviors indicates that we may need to target less educated cancer survivors to improve observed disparities and prevent further inequities in cigarette/e-cigarette smoking and physical inactivity. In addition, the associations of higher income with risky alcohol use observed in our study are comparable to a previous study that found higher income earners were more likely to be current or heavy drinkers, yet less likely to be cigarette smokers and physically inactive [5, 41].

We also observed that non-Hispanic White cancer survivors were more likely to be risky drinkers and current smokers (both cigarette and e-cigarette), and Black/African American cancer survivors were vulnerable to not meeting physical activity guidelines. Our findings are consistent with a recent study using US adults (*n* = 9761) in the National Alcohol Survey [60]. In our study, nearly two thirds of cancer survivors did not meet the physical activity guidelines, highlighting the need to better understand the barriers to achieving physical activity goals. For example, a lack of social support for the physical activity promotion was addressed as a main barrier among Black/African American women [76].

Addressing unhealthy behaviors in cancer survivors is important for improving outcomes in this population. Previously, it was reported that risky alcohol consumption and current cigarette smoking were associated with increased all-cause and cancer specific mortality [15–22, 77], while moderate physical activity was associated with improved all-cause and cancer specific mortality [29]. Although recent evidence showed that e-cigarette smoking could facilitate cancer progression and potentially exacerbate mental challenges, including depression, e-cigarette use is not often included in current tobacco product cessation policies [24–27, 78]. Thus, efforts and resources to support risky health behavior modifications, including e-cigarette cessation, should target the most vulnerable subgroups of cancer survivors that we identified in this study.

This study has some limitations. First, our study used self-report based cross-sectional survey data. Although it is nationally representative and validated resource, it carries inherited weakness of subjectivity originated from self-reporting. In addition, low response rate, 30% on average across the cycles from 1 to 4, could be a potential source of bias even though the data accounted for non-response. Moreover, the small sample size of current and former e-cigarette users limited our ability to identify high-risk subgroups. Further investigation with larger sample size will be necessary to understand the factors associated with e-cigarette smoking in cancer survivors. However, we are among the first studies to examine e-cigarette use among cancer survivors, reporting the prevalence and the associated factors of e-cigarette use. Alcohol consumption was not reported in HINTS 5 cycle 1, resulting in a smaller sample size than other outcomes (*n* = 1827). Lastly, our study cannot show the temporality of associations between unhealthy behaviors with sociodemographic and clinical characteristics. Hence, our findings are unable to take into consideration changes in health behaviors over time within cancer survivors. Despite these limitations, the strengths of the present study include the comprehensive investigation of unhealthy behaviors by sociodemographic and clinical characteristics among cancer survivors diagnosed with all types of cancer.

## Conclusion

Our findings suggest that cancer subgroups who are males, non-Hispanic Whites or Black/African American, without a college degree, not married, and with comorbidities or psychological distress were more likely to have risky health behaviors. Furthermore, we observed clusters of risky health behaviors, particularly cigarette smoking, e-cigarette use, and risky drinking. Our findings inform clinicians and program and policy makers of the subgroups of cancer survivors to target for health behavior interventions.

### Supplementary Information

Below is the link to the electronic supplementary material.

Supplementary file1 (XLSX 18 KB)

## Data Availability

The data for this study is publicly available at https://hints.cancer.gov/data/download-data.aspx.
